# Serum tryptophan decrease correlates with immune activation and impaired quality of life in colorectal cancer

**DOI:** 10.1038/sj.bjc.6600336

**Published:** 2002-06-05

**Authors:** A Huang, D Fuchs, B Widner, C Glover, D C Henderson, T G Allen-Mersh

**Affiliations:** Department of Surgery, Faculty of Medicine, Imperial College of Science, Technology and Medicine, Chelsea and Westminster Hospital, 369 Fulham Road, London SW10 9NH, UK; Department of Immunology, Faculty of Medicine, Imperial College of Science, Technology and Medicine, Chelsea and Westminster Hospital, 369 Fulham Road, London SW10 9NH, UK; Institute for Medical Chemistry and Biochemistry and Ludwig Boltzmann Institute of AIDS-Research, University of Innsbruck, Fritz Pregl Strasse 3, A-6020, Innsbruck, Austria

**Keywords:** tryptophan, quality of life, colorectal cancer, immune activation

## Abstract

Cancer-related indoleamine (2,3)-dioxygenase up-regulation by interferon-γ might influence quality of life by depleting serum tryptophan. We correlated serum tryptophan levels with immune activation and quality of life in patients with colorectal liver metastases. Venous blood was sampled from patients with primary colorectal cancer and from patients with metachronous colorectal liver metastases who completed quality of life and psychological questionnaires. Serum tryptophan, kynurenine, neopterin, interleukin 2 soluble receptor α (IL-2 sRα), soluble tumour necrosis factor receptor I (sTNF RI), interleukin 6, and C-reactive protein were measured. Liver metastasis volume was estimated by computerised tomography, and survival from blood sampling was noted. Sixty-six patients with colorectal cancer were studied (39 males; median age 66 years) of whom 25 had colorectal liver metastases only (17 males; median age 62 years; median liver metastasis volume 208 ml; median survival 234 days). Reduced serum tryptophan was significantly associated with Rotterdam Symptom Checklist physical symptom (*r*=−0.51, *P*=0.01) and Sickness Impact Profile (*r*=−0.42, *P*=0.04) scores, and correlated with increased serum neopterin (*r*=−0.36, *P*=0.003), IL-2 sRα (*r*=−0.51, *P*=0.01) and sTNF RI (*r*=−0.45, *P*=0.02) levels. Stepwise regression analyses suggested that serum tryptophan was an independent predictor of Rotterdam Symptom Checklist physical symptom (regression coefficient −20.78, *P*=0.01) and Sickness Impact Profile (regression coefficient −109.09, *P*=0.04) scores. The results supported a role for interferon-γ-mediated serum tryptophan decrease in cancer-induced quality of life deterioration.

*British Journal of Cancer* (2002) **86**, 1691–1696. doi:10.1038/sj.bjc.6600336 www.bjcancer.com

© 2002 Cancer Research UK

## 

One explanation for the deterioration in the quality of life (QoL) of colorectal cancer patients (Earlam *et al*, 1996) is an immune response to tumour-related products. In support of this, QoL impairment in colorectal liver metastasis (CLM) patients has been shown to correlate more closely with extent of immune activation than with liver metastasis volume or the serum level of the tumour marker carcinoembryonic antigen (CEA) (Allen-Mersh *et al*, 1998). In addition, animal studies suggest that immune activated cytokines induce sickness behaviour (Dantzer *et al*, 1998) that is similar to QoL impairment in humans (Bauhofer *et al*, 2001). However, a mechanism by which tumour-related immune activation could result in QoL deterioration has not been identified.

The Th1 immune response stimulates the release of interferon-γ which induces the enzyme indoleamine (2,3)-dioxygenase (IDO) (Murr *et al*, 2000). This increases degradation of tryptophan to kynurenine (Werner *et al*, 1989), thereby reducing serum tryptophan (Gasse *et al*, 1998) and releasing kynurenine which is then rapidly degraded (Denz *et al*, 1993). The neurotransmitter serotonin is synthesised from tryptophan, and decreased levels of tryptophan, serotonin and 5-hydroxyindoleacetic acid (the principal serotonin catabolite) can be associated with depression (Owens and Nemeroff, 1994; Smith *et al*, 1999). Treatment with selective serotonin reuptake inhibitors may alleviate this depression (DeVane, 1998), but serum tryptophan depletion can also result in transient, incomplete relapse (Smith *et al*, 1997). Although the contribution of reduced serum tryptophan to depression remains controversial, the finding of decreased tryptophan in both haematological (Denz *et al*, 1993) and non-haematological (Iwagaki *et al*, 1997) malignancies, suggests a mechanism by which cancer-related immune stimulation might influence QoL (Murr *et al*, 2000).

In this study, we measured serum tryptophan and kynurenine levels in patients with colorectal cancer, and correlated these levels with immune activation and QoL in CLM patients.

## PATIENTS AND METHODS

### Patients studied and blood sampling

All cancer patients had histologically-proven adenocarcinoma of the colon or rectum. Consecutive patients with primary colorectal cancer were studied prior to tumour resection, and showed no evidence of metastases on pre-treatment abdominal CT scan or chest radiograph. CLM patients had undergone primary colorectal cancer resection more than 3 months and less than 24 months previously, and had typical features of liver metastases on hepatic computerised tomographic (CT) scan, together with a rising serum CEA level. These patients had failed to respond to 1st line chemotherapy (5-fluorouracil and folinic acid) and were being considered for percutaneous liver metastasis cryotherapy (Huang *et al*, 2002). Patients were allocated to a ‘weight loss’ group if they had lost more than 1 kg in body-weight during the previous month and otherwise to a ‘stable weight’ group (Fordy *et al*, 1999). Patients with extra-hepatic metastases on abdominal or pelvic CT scan, or on thoracic radiograph, and patients receiving anti-depressant, cytotoxic or corticosteroid treatments were excluded.

Subjects with no history of cancer, undergoing herniorraphy or haemorrhoidectomy, were included as ‘no-cancer’ controls.

Ten millilitre of peripheral venous blood was sampled at 0800 h in the morning after overnight fasting, and the serum stored at −20°C until assayed.

### Serum tryptophan and kynurenine measurements

Serum free tryptophan and kynurenine concentrations were measured by reverse-phase high-pressure liquid chromatography after precipitation of protein with trichloroacetic acid. Tryptophan was measured by fluorescence detection at 285 nm excitation wavelength and 365 nm emission wavelength; kynurenine was measured by UV-absorption of 360 nm wavelength (Fuchs *et al*, 1990). The mean values from previous measurements – in 49 healthy individuals – within our laboratory (Widner *et al*, 1997) were: tryptophan 73.0 μmol l^−1^ (standard deviation [s.d.]±14.9), kynurenine 1.9 μmol l^−1^ (s.d.±0.6) and kynurenine/tryptophan ratio 26.9×10^−3^ (s.d.±8.1×10^−3^).

### Serum immune product measurements

Serum neopterin was measured using radioimmunoassay (double antibody technique). Normal serum value reported by the supplier (Brahms Diagnostica GmbH, Berlin, Germany) was: mean 5.4 nmol l^−1^ (s.d.±2.3) with an upper normal limit of 10 nmol l^−1^. Serum interleukin 2 soluble receptor α (IL-2 sRα), soluble tumour necrosis factor receptor I (sTNF RI) and interleukin 6 (IL-6) were measured using enzyme-linked immunosorbent assays. Normal serum values reported by the supplier (R&D Systems Europe Ltd., Abingdon, UK) were: IL-2 sRα, median 1346 ng l^−1^ (range 676–2132), sTNF RI, 1198 ng l^−1^ (749–1966), IL-6, 1.6 ng l^−1^ (0.4–10.1). The upper value in each range was taken as the upper limit of normal. The IL-2 sRα assay coefficients of variation were<6.1% (intra-assay) and<7.2% (inter-assay), and those for other assays were within a similar range. Serum C-reactive protein (CRP) was measured by an automated immunoturbidimetric assay on an Olympus AU600 analyser (Olympus Diagnostic Systems, Eastleigh, UK), and a serum CRP concentration of 10 mg l^−1^ was taken as the upper limit of normal.

### Liver metastasis volume, survival and QoL measurements in CLM patients

Within 3 weeks of blood sampling, liver metastasis volume was measured by CT scan as previously described (Dworkin *et al*, 1995). Survival was measured in days from the date of blood sampling.

On the day of blood sampling, patients completed the following QoL and psychological questionnaires: Rotterdam Symptom Checklist (RSC) (de Haes *et al*, 1990), Hospital Anxiety and Depression scale (HAD) (Zigmond and Snaith, 1983), and the Sickness Impact Profile (SIP) (Bergner *et al*, 1981).

### Statistical analysis

Differences between groups in serum tryptophan, kynurenine and kynurenine/tryptophan ratio were assessed by Mann–Whitney *U*- (MWU) test. Spearman rank correlation (Langley, 1979) and stepwise multiple regression analysis (Dixon, 1992a) were used for statistical analyses as indicated. Tumour volume and serum levels were not normally distributed and the interquartile ranges (iqr, 25th to 75th percentile) around the median were therefore used; natural logarithms were also used to ensure normality before the model was fitted in regression. Similarly, the natural logarithm of survival was used as an independent variable in stepwise multiple regression analysis when the QoL score was the dependent variable. Survival was included as an independent variable in case other variables were dependent on it. The regression analyses excluded patients who were still alive (four out of 25 patients) at the completion of follow-up. Variables on these four patients were within the interquartile ranges and therefore unlikely to distort findings by their exclusion. Multivariate analyses assessing whether the levels of serum immune products, tryptophan and kynurenine contributed to variability in QoL scores used both natural logarithms of continuous and binary transformed (above/below upper and lower limits of normal/percentile as appropriate) variables. *P* values below 0.05 were considered statistically significant.

The study was approved by the Chelsea and Westminster Hospital Research Ethics Committee and patients gave informed consent before participation in the study.

## RESULTS

### Patients

One hundred and three patients [66 colorectal cancer patients (39 males; median age 66 years, iqr 61–73) and 37 ‘no-cancer’ controls (13 males; median age 69 years, iqr 64–76)] were studied.

Serum immune product levels were measured in 25 of the colorectal cancer patients who had liver metastases (17 males; median age 62 years, iqr 56–64) and who had also completed QoL questionnaires (Table 1

**Table 1:**
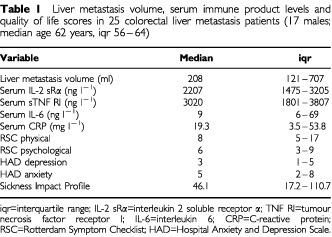
Liver metastasis volume, serum immune product levels and quality of life scores in 25 colorectal liver metastasis patients (17 males; median age 62 years, iqr 56–64)

). Median survival from blood sampling for liver metastasis patients was 234 days (iqr 165–387), with four of the liver metastasis patients surviving at the end of follow-up (25, 28, 32, and 35 months from blood sampling).

### Serum tryptophan, kynurenine, and neopterin levels

The serum kynureinine/tryptophan ratio and neopterin were significantly increased and serum tryptophan significantly reduced in colorectal cancer compared with control patients (Table 2

**Table 2:**
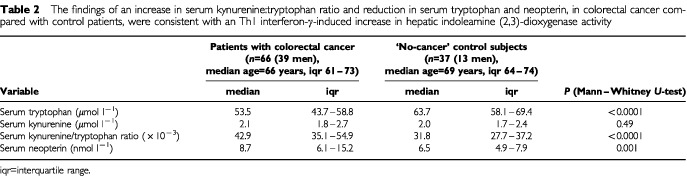
The findings of an increase in serum kynurenine:tryptophan ratio and reduction in serum tryptophan and neopterin, in colorectal cancer compared with control patients, were consistent with an Th1 interferon-γ-induced increase in hepatic indoleamine (2,3)-dioxygenase activity

). There was a significant correlation between the kynurenine/tryptophan ratio and serum neopterin in both colorectal cancer patients (Spearman rank correlation, *r*=0.63, *P*<0.0001, 95% confidence interval [95% CI]=0.45 to 0.75, *n*=66) and in ‘no-cancer’ controls (*r*=0.54, *P*=0.0005, 95% CI=0.26 to 0.74, *n*=37).

There was a significant reduction (MWU, *P*=0.03) in serum tryptophan level in colorectal cancer patients with liver metastases (median 48.5 μmol l^−1^, iqr 43.3–56.4) compared with those with primary tumour but no metastases (median 57.0 μmol l^−1^, iqr 48.8–64.4). Serum tryoptophan level was significantly lower (MWU, *p*=0.01) in liver metastasis patients who had lost >1 kg in body weight during the preceding month (*n*=3, median 33.1 μmol l^−1^, iqr 30.1–34.5) compared with those who had not (*n*=16, median 45.3 μmol l^−1^, iqr 43.2–53.1). There were no significant correlations between serum tryptophan, and liver metastasis volume or extent of survival in liver metastasis patients.

### Relation between serum tryptophan and QoL in liver metastasis patients

**Figure 1:**
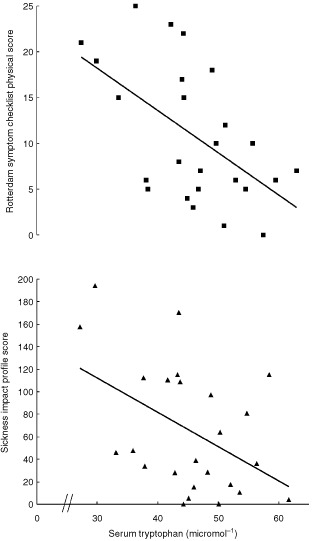
Reduced serum tryptophan correlated significantly (Spearman rank correlation) with quality of life deterioration in 25 patients with colorectal liver metastases only (upper, Rotterdam Symptom Checklist physical score, *r*=−0.51, *P*=0.01, 95% CI=−0.75 to −0.14; lower, Sickness Impact Profile score, *r*=−0.42, *P*=0.04, 95% CI=−0.70 to −0.03)

There were significant correlations (Spearman rank correlation) between reduced serum tryptophan level, and increased RSC physical and SIP scores (Figure 1

), but not HAD depression, HAD anxiety or RSC psychological scores. Stepwise multiple regression analysis, including survival, tumour volume and serum immune product levels as independent variables, selected serum tryptophan as the most significant independent predictor of RSC physical symptom score, and a significant independent predictor of SIP score (Table 3

**Table 3:**
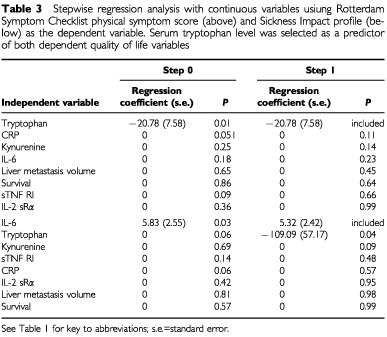
Stepwise regression analysis with continuous variables usiung Rotterdam Symptom Checklist physical symptom score (above) and Sickness Impact profile (below) as the dependent variable. Serum tryptophan level was selected as a predictor of both dependent quality of life variables

). A similar result was obtained using binary variables (normal/elevated serum levels). If included in the regression analysis, serum neopterin was selected before serum tryptophan level. Serum tryptophan was not selected as an independent predictor of other QoL variables.

### Serum tryptophan and immune products in liver metastasis patients

Reduced serum tryptophan significantly correlated with serum IL-2 sRα (Spearman rank correlation, *r*=−0.51, *P*=0.01, 95% CI=−0.76 to −0.15, *n*=25) and sTNF RI (*r*=−0.45, *P*=0.02, 95% CI=−0.72 to −0.07, *n*=25), but not with IL-6 or CRP.

## DISCUSSION

Although fatigue or depression affects most patients with cancer (Stone *et al*, 2000), the underlying mechanisms are still poorly understood (Curt, 2001). The associations identified in the present study do not establish cause and effect, but they are consistent with QoL deterioration being influenced by an interferon-γ-mediated reduction in serum tryptophan. Multiple analyses were performed on a limited number of patients in the present study. However, the statistical significance of tryptophan depletion in colorectal cancer (Table 2), and of the association between serum tryptophan level and QoL in regression analysis, remained after Bonferroni correction (Dixon, 1992b).

Activated T lymphocytes release interferon-γ which stimulates macrophage production of neopterin. As a result there is a close correlation between interferon-γ activity and serum neopterin level (Murr *et al*, 1999). The correlations between serum neopterin and the serum kynurenine/tryptophan ratio in both colorectal cancer patients and in ‘no cancer’ controls, and the rise in the kynurenine/tryptophan ratio in colorectal cancer patients suggested an interferon-γ-induced increase in IDO activity (Murr *et al*, 2000) among colorectal cancer patients. Increased serum kynurenine would not be expected because of rapid kynurenine metabolism (Denz *et al*, 1993). The negative correlation of the serum tryptophan level with IL-2 sRα and sTNF RI was compatible with a Th1 immune response (Mills *et al*, 1993; Diez-Ruiz *et al*, 1995) releasing interferon-γ to induce tryptophan degradation (Murr *et al*, 2000).

Cachexia studies in pancreatic cancer have suggested (Preston *et al*, 1998) that a tumour-associated acute phase protein response induces fibrinogen synthesis at the expense of skeletal muscle reserves. The greater aromatic amino acid (mostly tryptophan) content in fibrinogen compared with skeletal muscle proteins, may then result in tryptophan depletion. Unlike pancreatic cancer, significant weight loss in colorectal cancer is usually associated with a large tumour burden (Fordy *et al*, 1999). Our results suggested a substantial serum tryptophan reduction (48% compared with ‘no-cancer’ controls), in the small number of colorectal liver metastasis patients with a history of more than 1 kg weight loss over the previous month. However, the absence of a significant association between serum CRP (an indicator of the extent of acute phase protein response) and serum tryptophan level, and the finding of reduced serum tryptophan in the majority of colorectal cancer patients without weight loss, did not suggest that our results could entirely be explained by muscle wasting associated with tryptophan depletion. It is possible that both an interferon-γ-induced increase in IDO activity and acute phase protein synthesis, were part of a host response to colorectal carcinoma that depleted the serum of tryptophan.

We cannot explain the difference between our results and some earlier reported findings of elevated levels of plasma free tryptophan in animals (Krause *et al*, 1979) and patients (Cangiano *et al*, 1990) with cancer – particularly those with anorexia (Chance *et al*, 1983; Fanelli *et al*, 1986). In the present study, laboratory analysis was carried out on mixed batches of control and cancer patient samples without knowledge of the patient source. The results showed a statistically significant 16% lowering of serum tryptophan among colorectal cancer patients (24% in liver metastasis patients) compared with ‘no cancer’ controls, and this was compatible with other more recent results reported for patients with a variety of cancer types (Denz *et al*, 1993; Giusti *et al*, 1996; Iwagaki *et al*, 1997).

Studies in non-cancer patients with depression suggest that rapid tryptophan depletion to roughly 20% of normal plasma levels (Delgado *et al*, 1990; Smith *et al*, 1999) can induce a relapse of depression (Moore *et al*, 2000). Acute tryptophan depletion of a similar magnitude in healthy volunteers has been associated with impairment of long-term memory (Riedel *et al*, 1999). The cognitive effect of a chronic 10–20% serum tryptophan lowering (probably double this in patients with cachexia), is unknown. The selection in regression analysis of serum neopterin as the strongest independent predictor of RSC physical and SIP scores was consistent with an association between interferon-γ up-regulation and adverse QoL. The elimination of serum tryptophan level from QoL regression analyses where serum neopterin was included, suggested that serum neopterin and tryptophan levels provided similar information about QoL prediction, and this would be expected from the metabolic relationship between interferon-γ up-regulation and IDO-mediated tryptophan degradation. When serum neopterin was excluded from the regression analysis, then reduced serum tryptophan was selected as an independent predictor of deterioration in RSC physical and SIP scores (Table 3). These findings were consistent with an interferon-γ-mediated serum tryptophan decrease leading to QoL impairment in cancer patients.

In addition to serum neopterin and tryptophan, serum IL-2 sRα and TNF RI levels were also selected as independent predictors of QoL deterioration. Thus the results suggested that a variety of immune-related factors influenced QoL in CLM patients (Allen-Mersh *et al*, 1998). Immune cytokines have overlapping functions and act synergistically with each other in a concerted manner, so that small alterations in particular combinations of cytokines can have larger effects. Cytokines are capable of a wide array of direct interactions – for example with central nervous system (Maes *et al*, 1995; Zalcman *et al*, 1994), liver, and muscle receptors (Beutler *et al*, 1985) – as well as mediating wasting (Fordy *et al*, 1999) in colorectal cancer.

Although tryptophan supplementation has been used to treat depression (DeVane, 1998), such an approach would be unlikely to be effective where there is cancer-related IDO induction, because this would increase IDO-associated neurotoxic metabolites – such as quinolinic acid (Saito *et al*, 1992). Serum tryptophan depletion is thought to influence mood in healthy subjects by the impairment of central serotonergic neurotransmission (Moore *et al*, 2000). Selective serotonin reuptake inhibitors (SSRI) have been reported to improve depression in advanced cancer (Holland *et al*, 1998), and in melanoma treated with interferon α (Musselman *et al*, 2001) since the latter also upregulates IDO (Taylor and Feng, 1991). However, these SSRI antidepressant effects might not depend on IDO-mediated serum tryptophan reduction, and studies of the effect of IDO inhibitors (Yuan *et al*, 1998) on cancer-related QoL deterioration would be needed to confirm the mechanism suggested by the present findings.
